# Interleukin-1α Activity in Necrotic Endothelial Cells Is Controlled by Caspase-1 Cleavage of Interleukin-1 Receptor-2: IMPLICATIONS FOR ALLOGRAFT REJECTION*

**DOI:** 10.1074/jbc.M115.667915

**Published:** 2015-08-31

**Authors:** Laura C. Burzynski, Melanie Humphry, Martin R. Bennett, Murray C. H. Clarke

**Affiliations:** From the Division of Cardiovascular Medicine, University of Cambridge, Level 6, Box 110, Addenbrooke's Hospital, Cambridge CB2 0QQ, United Kingdom

**Keywords:** calpain, caspase, endothelial cell, inflammation, interleukin 1 (IL-1), necrosis (necrotic death), transplantation, vascular biology, vascular smooth muscle cells, interleukin-1 receptor-2

## Abstract

Inflammation is a key instigator of the immune responses that drive atherosclerosis and allograft rejection. IL-1α, a powerful cytokine that activates both innate and adaptive immunity, induces vessel inflammation after release from necrotic vascular smooth muscle cells (VSMCs). Similarly, IL-1α released from endothelial cells (ECs) damaged during transplant drives allograft rejection. However, IL-1α requires cleavage for full cytokine activity, and what controls cleavage in necrotic ECs is currently unknown. We find that ECs have very low levels of IL-1α activity upon necrosis. However, TNFα or IL-1 induces significant levels of active IL-1α in EC necrotic lysates without alteration in protein levels. Increased activity requires cleavage of IL-1α by calpain to the more active mature form. Immunofluorescence and proximity ligation assays show that IL-1α associates with interleukin-1 receptor-2, and this association is decreased by TNFα or IL-1 and requires caspase activity. Thus, TNFα or IL-1 treatment of ECs leads to caspase proteolytic activity that cleaves interleukin-1 receptor-2, allowing IL-1α dissociation and subsequent processing by calpain. Importantly, ECs could be primed by IL-1α from adjacent damaged VSMCs, and necrotic ECs could activate neighboring normal ECs and VSMCs, causing them to release inflammatory cytokines and up-regulate adhesion molecules, thus amplifying inflammation. These data unravel the molecular mechanisms and interplay between damaged ECs and VSMCs that lead to activation of IL-1α and, thus, initiation of adaptive responses that cause graft rejection.

## Teaser

**Background:** IL-1α released from damaged endothelial cells (ECs) drives inflammation and chronic graft rejection. How ECs activate IL-1α is unknown.

**Results:** Generation of active IL-1α in ECs requires caspase-1 proteolysis of an inhibitory complex that releases IL-1α and enables calpain cleavage to the mature cytokine.

**Conclusion:** IL-1α is controlled post-translationally in ECs.

**Significance:** Multiple targets exist to prevent IL-1α activation during disease.

## Introduction

Chronic transplant allograft rejection is the leading cause of late graft failure and mortality. Rejection is most commonly caused by graft arteriosclerosis (chronic allograft vasculopathy), which occurs due to a host immune alloresponse directed against the graft blood vessels (1). Allograft vasculopathy is characterized by progressive stenosis of vessels throughout the organ due to deposition of extracellular matrix, VSMC^2^ proliferation, and constrictive remodeling that occurs over months to years. Vessel stenosis leads to ischemia, which is thought to be the primary cause of parenchymal fibrosis within the tissue, loss of function, and graft failure. Late graft failure occurs at a rate of 5% per year post transplantation in the main solid organ transplants (kidney, heart, liver) (1) and is currently untreatable. Graft ECs expressing MHC molecules are a major target of this alloresponse, causing activation of CD4 memory T cells and IFNγ production that is vital for arteriosclerosis (2). In addition, other non-immune mechanisms also modulate the development of arteriosclerosis.

Grafts are exposed to both physical and ischemic injuries during the pre- and perioperative period. In particular, cadaveric donor grafts fail at accelerated rates, which may be due to the greater injury they receive. The two main theories of why injury drives graft failure are the “burden of injury hypothesis” (3) and the “immune modulation hypothesis” (1). The former postulates that grafts are exposed to multiple other injuries such as periods of acute rejection, chronic rejection, and other insults and can only tolerate a finite amount before the graft fails. Thus, grafts exposed to more perioperative injury simply exceed this limit earlier. The latter proposes that early graft injury somehow alters a graft such that it interacts with the host's immune system in a way that accelerates rejection. The two theories are likely linked, with graft damage driving cell proliferation in the vessel wall as a typical response to injury and injured necrotic cells acting as a potent source of damage-associated molecular patterns (DAMPs) that directly stimulate innate and adaptive immune cells. DAMPs are typically molecules that are exclusively intracellular under normal conditions and, thus, their presence outside the cytosol indicates cell damage and/or necrosis (4). Identified DAMPs include high-mobility group box 1, ATP, heat shock proteins, actin, uric acid, DNA, and IL-1α (5). IL-1α is released from necrotic ECs and is particularly important in graft rejection as it drives IL-17 and IFNγ production from infiltrating memory CD4+ T cells (6, 7), which are two cytokines of key significance in enhanced alloimmunity.

IL-1α binds to the type 1 IL-1 receptor (IL-1R1) and recruits IL-1 receptor accessory protein to form a complex that interacts with the signaling adapter MyD88 (8). Following a phospho-signaling cascade, NF-κB is activated, inducing expression of multiple proinflammatory genes (8). IL-1α is near universally expressed and resides in the cytosol as a 33-kDa precursor that is reported to be fully biologically active, and thus release from damaged cells enables it to act as a universal danger signal or “alarmin.” However, recent work has shown that pro-IL-1α has far less cytokine activity than the calpain-cleaved mature form and acts as a partial agonist due to low binding affinity for IL-1R1 (9, 10). Furthermore IL-1α activity after necrosis is regulated in a cell type-specific manner with most cells unable to cleave IL-1α upon necrosis and, therefore, contain no IL-1α activity (10). IL-1α released from damaged ECs drives the T helper 17 cell (Th17) responses that induce vessel arteriosclerosis and subsequent allograft rejection (6, 7), suggesting that ECs must be able to activate pro-IL-1α. However, whether ECs constitutively express IL-1α, what causes cleavage and/or activation of IL-1α and the mechanism controlling this is not known. We find that IL-1α activity is regulated via a novel mechanism involving TNFα- and IL-1-induced caspase activity that cleaves IL-1R2, causing IL-1α dissociation that enabled subsequent processing by calpain.

## Experimental Procedures

### 

#### 

##### Tissue Culture

All material was from Sigma unless stated otherwise. Human umbilical vein ECs (HUVECs) were cultured in large vessel endothelial cell media with supplements (all TCS Cellworks), human aortic VSMCs and HeLa cells were cultured in DMEM, 10% FCS, 10 units/ml penicillin, 10 mg/ml streptomycin, 5 mg/ml l-glutamine, and all were passaged at 80% confluence. EL4 and THP1 cells were cultured in RPMI, 10% FCS, 10 units/ml penicillin, 10 mg/ml streptomycin, 5 mg/ml l-glutamine, 2.3 μm β-mercaptoethanol and maintained between 4–10 × 10^5^/ml. Where indicated HUVEC cells were pretreated for 16 h with IL-1β (10 ng/ml) or TNFα (10 ng/ml; both Peprotech) with and without benzyloxycarbonyl-VAD-fluoromethyl ketone (100 μm, Bachem) or benzyloxycarbonyl-YVAD-fluoromethyl ketone (20 μm, BioVision). Necrotic lysates were made by resuspending cells in serum-free DMEM with and without calpeptin (30 μm; Enzo) and freeze/thawing 3 times in liquid N_2_ before removal of insoluble material by centrifugation (1 × 10^4^ g; 3 min). Necrotic lysates were made at 1 × 10^5^ (HUVECs), 15 × 10^4^ (THP1 and HeLa), or 5 × 10^4^ (VSMCs) cells per 72 μl of serum-free DMEM. Western blot lysates were made with 600,000 cells per 24 μl of media. To detect IL-1α-specific activity, control VSMCs were adhered overnight and then incubated in low serum (1%) media for ∼24 h, whereas HeLa cells and HUVECs were only adhered overnight in full media. Media were replaced along with treatments as indicated and incubated for 6 h. EL4 cells were washed and plated in serum-free media along with treatments and incubated for 24 h. Conditioned media was collected and clarified, and cytokines were assayed as below. Specific IL-1α activity was inferred with a neutralizing antibody (2 μg/ml; Peprotech) added during the 6-h incubation. HUVECs were transfected by nucleofection (VPB-1002; program A034; Lonza). 250 × 10^3^ cells were transfected with wild type or mutant IL-1R2 or empty vector (pcDNA 3.1; 2 μg; Invitrogen) along with pmaxGFP (0.2 μg; Lonza) before electroporation. Cells were transferred into full media and incubated for 2 days before use.

##### Cytokine Measurement

Clarified supernatants were analyzed by plate ELISA for IL-1α and IL-2 (both Peprotech), or cytometric bead ELISA for IL-6, IL-8, and MCP-1 (eBioscience) as per the manufacturer's instructions. Plates were measured at 450 nm on a spectrophotometer (Gen5) with the data analyzed using a 4-parameter logistic standard curve. Beads were measured by cytometry (Accuri C6), and data were analyzed with FlowCytomix Pro (eBiosciences).

##### RT-PCR and Quantitative PCR

RNA was isolated using TRIzol reagent and DNase-treated (Ambion) before reverse transcription with MMLV and oligo(dT) (Promega). Quantitative PCR used TaqMan probe/primers with AmpliTaq gold (Life Technologies) in a RotorGene thermocycler (Corbett). Analysis utilized standard curves with specific expression level normalized to GUSB and TBP. IL-1R2 transcript was assessed by RT-PCR using the primers: soluble IL-1R2 forward (TGGCACCTACGTCTGCACTA) and reverse (TGTCTCCAAAAGGAAGAGCGA); membrane IL-1R2 forward (ACACGGATGTGGGCCCAGGA) and reverse (GTGAAAGTGGGGCCAGCACA); GAPDH forward (TGTTGCCATCAATGACCCCTT) and reverse (CTCCACGACTGACTCAGCG).

##### Western Blotting

Western Blotting was performed as previously described with lysis of cells directly in Laemmli buffer, SDS-PAGE, and transfer onto PVDF membrane. After blocking (5% milk), membranes were incubated (4 °C; 16 h) with IL-1α pAb (1:500; Peprotech), IL-1R2 pAb (1:250; R&D), or β-actin mAb (1:5000; Sigma) before washing (PBS/Tween) and incubation (room temperature for 1 h) with anti-rabbit HRP (1:2000; GE Healthcare) or anti-goat HRP (1:2000; Jackson ImmunoResearch). After washing membranes were visualized with ECL reagent (Amersham Biosciences) and x-ray film (Fujifilm).

##### IL-1R2 Protection Assay

Necrotic HUVEC lysates from either control or IL-1β-treated cells were incubated with increasing amounts of His-tagged pro-IL-1α (0–250 ng) and incubated (37 °C; 1 h) before the addition of 3× Laemmli buffer, boiling, and analysis by Western blotting.

##### Calpain Activity

Calpain activity was assayed using the luminescent CalpainGlo kit (Promega). Test sample and reagents were mixed 50:50 and incubated (room temperature; 10 min) before analysis on a luminometer (Turner Biosystems).

##### Immunofluorescence and Proximity Ligation Assay

HUVECs adhered to coverslips were treated as indicated before fixation with formaldehyde (2%; room temperature; 15 min) and then methanol (10 min; −20 °C) before washing (PBS). Cells were blocked with BSA (1%; 1 h) before incubation (4 °C; 16 h) with IL-1α pAb (1:100; Aviva) and IL-1R2 mAb (1:40; R&D) in a humidified chamber. After washing (0.05% Tween, PBS), coverslips were either stained with Duolink probes as per the manufacturer's instructions (OLink Bioscience) along with a probe-only control or AlexaFluor goat anti-rabbit 488 (IL-1α) and goat anti-mouse 568 (IL-1R2) (both 1:500; 1 h) before mounting in ProLong Gold (all Invitrogen). Imaging was performed with a BX51 microscope (Olympus) with CellD imaging software (SIS).

##### Flow Cytometry

HUVECs in suspension were fixed in formaldehyde (2%; 10 min), washed (PBS) by centrifugation (220 × *g*; 5 min), resuspended into methanol (−20 °C; 10 min), washed, and blocked in BSA (1%; 1 h). Cells were incubated (30 min; room temperature) with IL-1R2 mAb (1:40; R&D) or IL-1α pAb (1:100; Aviva), washed, and incubated with AlexaFluor goat anti-rabbit 488 (IL-1α) or goat anti-mouse 568 (IL-1R2) (both 1:500; 40 min). A matched isotype and secondary control was performed for each HUVEC condition and subtracted from the mean fluorescent intensities values shown. Washed cells were resuspended in 200 μl of 1% BSA, PBS and analyzed by flow cytometry. For intracellular staining, saponin (0.1%) was added to buffers throughout to permeabilize cells, changing to 1% BSA/PBS before analysis by flow cytometry.

##### Statistics

Data are presented as the mean ± S.E. unless otherwise stated. All assays that produced continuous data, with the exception of flow cytometry, were performed in duplicate. *n* = an individual experimental replicate. Parametric tests were employed for analysis of continuous data, conducted using a one-way, two-tailed analysis of variance (Excel). Significance is as stated, but always *p* < 0.05. Non-significant data were considered anything where *p* > 0.05. All Westerns blots presented are representative of three separate experiments.

## Results

### 

#### 

##### IL-1α Activity in Necrotic Endothelial Cells Is Controlled Independently of Protein Level

**FIGURE 1.**
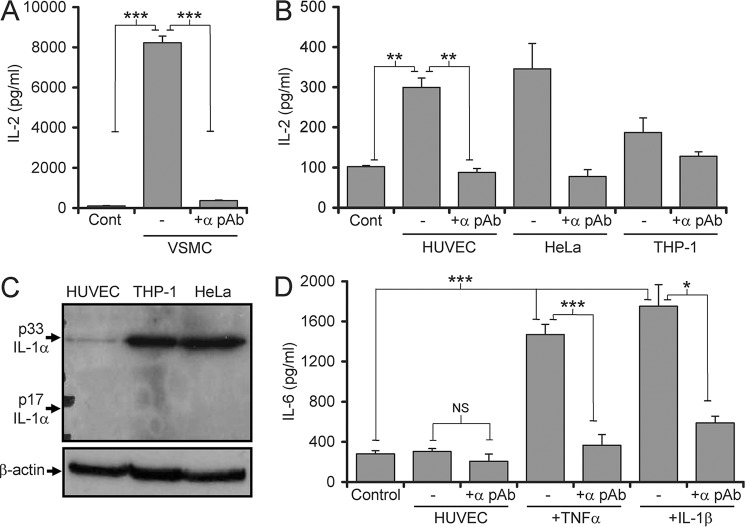
**IL-1α activity in necrotic endothelial cells is controlled independently of protein level.** *A* and *B*, IL-1-dependent IL-2 production by EL4 cells incubated with necrotic VSMC lysates (*A*) or necrotic cell lysates as indicated (*B*) with and without neutralizing IL-1α antibody (α *pAb*). *C*, Western blot for IL-1α or β-actin in whole cell lysates of cell-types indicated. *D*, IL-1-dependent IL-6 production by HeLa cells incubated with necrotic HUVEC lysates pretreated as indicated with and without IL-1α pAb. Data represent the mean ± S.E. of *n* = 3 (*A* and *B*) and ≥5 (*D*). *, *p* ≤ 0.05; **, *p* ≤ 0.01; ***, *p* ≤ 0.001.

IL-1α derived from injured donor ECs is required for intimal T cell recruitment and IL-17 production in graft allopathy (6, 7), suggesting that ECs must be able to activate IL-1α upon necrosis. We first examined necrotic HUVEC lysates for IL-1α activity using an EL4 cell bioassay in which IL-2 is produced in an IL-1-dependent manner. EL4 cells display very high sensitivity to IL-1, responding to femtomolar concentrations. Treatment of EL4 cells with necrotic VSMC lysates as a positive control (10) resulted in high levels of IL-2 production (∼8000 ± 200 pg/ml) (Fig. 1*A*), which was shown to be IL-1α-dependent with a neutralizing antibody. However, necrotic HUVEC lysates induced minimal IL-2 release, similar to HeLa and THP1 necrotic lysates (∼300 ± 25 pg/ml) (Fig. 1*B*). HeLa and THP-1 cells have previously been shown to contain negligible IL-1α activity after necrosis (10), suggesting that ECs are unable to activate IL-1α upon necrosis. However, despite comparable levels of IL-1α activity between the cell types, Western blot showed that HUVECs contain ∼10% of the IL-1α protein observed in THP1 and HeLa cells (Fig. 1*C*). This indicates that IL-1α protein expression in HUVEC necrotic lysates is not predictive of IL-1α activity, suggesting that another mechanism regulates IL-1α activity in ECs. Due to the high sensitivity of the EL4 bioassay, IL-1α activity in necrotic HUVEC lysates was reassessed using HeLa cells, which release IL-6 more linearly in response to IL-1 (10). Necrotic HUVEC lysates induced similar activity to the negative control (Fig. 1*D*). However, pretreatment of ECs with TNFα or IL-1β before making necrotic lysates resulted in a large increase in IL-1α-specific activity. Together these data suggest that unstimulated HUVECs contain minimal amounts of IL-1α activity but that this can be increased by activation of the cell before necrosis.

##### Increased IL-1α Activity in Activated ECs Occurs Due to IL-1α Cleavage by Calpain

**FIGURE 2.**
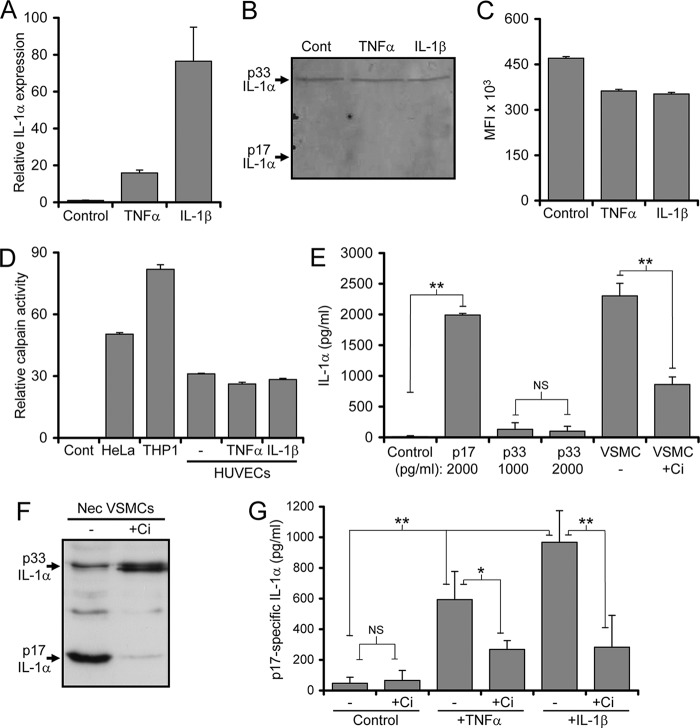
**Increased IL-1α activity in activated ECs occurs due to IL-1α cleavage by calpain.** *A* and *B*. level of IL-1α transcript relative to GusB by quantitative PCR (*A*) or protein by Western blot in whole cell lysates of HUVECs pretreated as indicated. *C*, mean fluorescent intensities (*MFI*) of HUVEC cells, pretreated as indicated, stained for IL-1α, and analyzed by flow cytometry. *D*, relative level of calpain activity using a substrate assay in necrotic lysates of the cell types treated as indicated. *E*, ELISA analysis of recombinant mature (*p17*) and pro-IL-1α (*p33*) at the concentrations indicated (pg/ml) and endogenous IL-1α within necrotic VSMC lysates made with and without calpain inhibition (+*Ci*). *F*, Western blot for IL-1α showing calpeptin (*Ci*) prevents processing of p33 to p17. *G*, mature IL-1α (*p17*) content by ELISA of necrotic HUVEC lysates pretreated as indicated and made with and without calpain inhibition (+*Ci*). Data represent the mean ± S.E. of *n* = 2 (*A* and *C*), 3 (*E*), and 4 (*F*); *, *p* ≤ 0.05, **, *p* ≤ 0.01; *NS* = not significant.

The increase in IL-1α activity seen in necrotic HUVECs after IL-1 or TNFα treatment could be due to increased IL-1α protein, increased IL-1α cleavage, or loss of an IL-1 antagonist. We, therefore, examined IL-1α mRNA in HUVECs treated with TNFα or IL-1. Although activated HUVECs had increased IL-1α transcripts compared with control cells (Fig. 2*A*), IL-1α protein levels were similar by Western blotting (Fig. 2*B*) or intracellular cytokine staining and flow cytometry (Fig. 2*C*), confirming that IL-1α activity is not correlated to IL-1α protein expression. Calpain cleavage of pro-IL-1α during necrosis significantly increases activity. As necrotic EC lysates contain calpain activity comparable with other cell types that were not altered by TNFα or IL-1β treatment (Fig. 2*D*), we investigated whether EC activation resulted in more IL-1α processing using a cleaved IL-1α-specific ELISA (Fig. 2, *E* and *F*). Pretreatment of ECs with TNFα or IL-1β before necrosis significantly increased cleaved IL-1α compared with control (Fig. 2*G*), which was inhibited by the calpain-specific inhibitor calpeptin (Fig. 2*G*). Thus, the increased IL-1α activity in necrotic EC lysates after activation is due to increased IL-1α cleavage and not increased IL-1α protein.

##### IL-1α Binding to IL-1R2 Prevents Calpain Cleavage and Controls Cytokine Activity in ECs

**FIGURE 3.**
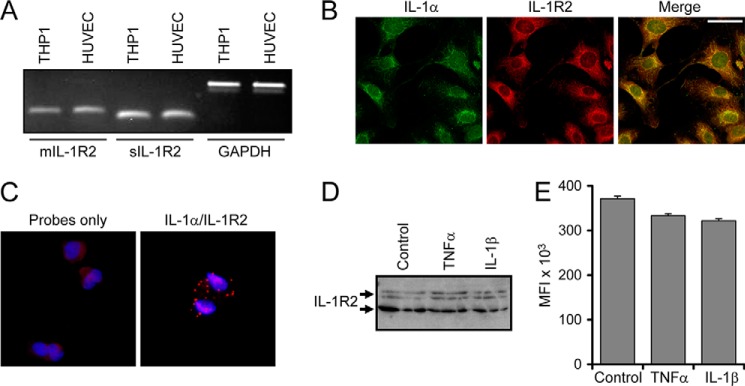
**IL-1α binding to IL-1R2 prevents calpain cleavage and controls cytokine activity in ECs.** *A*, RT-PCR for membrane (*mIL-1R2*) and soluble (*sIL-1R2*) IL-1R2 and GAPDH transcript within THP1 and HUVEC cells. *B*, dual immunofluorescence for pro-IL-1α and IL-1R2 in control HUVECs. *Scale bars* represent 50 μm. *C*, proximity ligation assay (DuoLink) showing association between IL-1α and IL-1R2 in control HUVEC cells. *D*, Western blot for IL-1R2 in whole cell lysates of HUVECs pretreated as indicated. *E*, mean fluorescent intensity (*MFI*) minus isotype control of HUVEC cells, pretreated as indicated, stained for IL-1R2, and analyzed by flow cytometry. Data represent the mean ± S.E. of *n* = 2.

IL-1α activity can be regulated by an intracellular form of IL-1R2 that protects pro-IL-1α from calpain cleavage (10). HUVECs expressed transcripts for both the membrane-bound and soluble forms of IL-1R2 (Fig. 3*A*), whereas immunofluorescence revealed pro-IL-1α and IL-1R2 to be highly co-localized (Fig. 3*B*). Although co-localization by immunofluorescence suggests association, it does not prove binding. However, an *in situ* proximity ligation assay (DuoLink) revealed that IL-1α and IL-1R2 are physically associated in control ECs (Fig. 3*C*). As IL-1R2 binding protects IL-1α from calpain cleavage, a general reduction in the steady state level of IL-1R2 protein could allow more processing and activation of IL-1α upon necrosis. However, TNFα or IL-1β treatment did not affect IL-1R2 protein levels on Western blotting (Fig. 3*D*) or flow cytometry (Fig. 3*E*).

##### EC Activation Reduces the Interaction between IL-1α and IL-1R2

**FIGURE 4.**
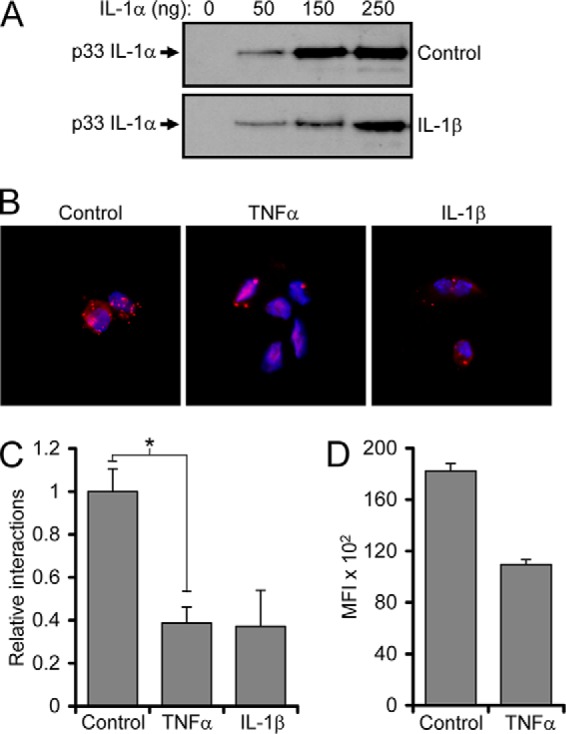
**EC activation reduces the interaction between IL-1α and IL-1R2.** *A*, Western blot for IL-1α in necrotic HUVEC lysates, pretreated as indicated, and incubated with increasing quantities of exogenous pro-IL-1α (p33). *B*, proximity ligation assay (DuoLink) showing association between IL-1α and IL-1R2 in HUVEC cells pretreated as indicated. *C* and *D*, relative number of interactions between IL-1α and IL-1R2 in HUVECs pretreated as indicated, assessed by DuoLink and quantified by blinded counting (*C*) and flow cytometry (*D*). *MFI*, mean fluorescent intensity. Data represent the mean ± S.E. of *n* = 2; *, *p* ≤ 0.05.

Most cell types have excess IL-1R2 that can protect exogenously added pro-IL-1α from calpain cleavage. Thus, by incubating necrotic EC lysates with increasing concentrations of exogenous pro-IL-1α and measuring the amount of uncleaved pro-IL-1α, we can determine the amount of “competent” IL-1R2. This showed that necrotic lysates from HUVECs prestimulated with IL-1β were less able to protect exogenous pro-IL-1α against cleavage compared with control lysates (Fig. 4*A*). This supports that activated ECs have more cleaved IL-1α (Fig. 2*G*) because IL-1R2 is less able to protect it from calpain cleavage. To confirm this, IL-1α/IL-1R2 association was quantified with DuoLink, which visually showed decreased association after TNFα and IL-1β treatment (Fig. 4*B*). Blinded interaction counts per cell confirmed this decrease (Fig. 4*C*), as did DuoLink performed in suspension quantified by FACs (Fig. 4*D*). Together these data suggest that IL-1α activity increases in pretreated necrotic EC lysates due to a reduction in IL-1R2/IL-1α association that subsequently allows IL-1α processing by calpain to the more active mature form.

##### Caspase-1 Cleavage of IL-1R2 Enables Induction of IL-1α Activity in Stimulated ECs

**FIGURE 5.**
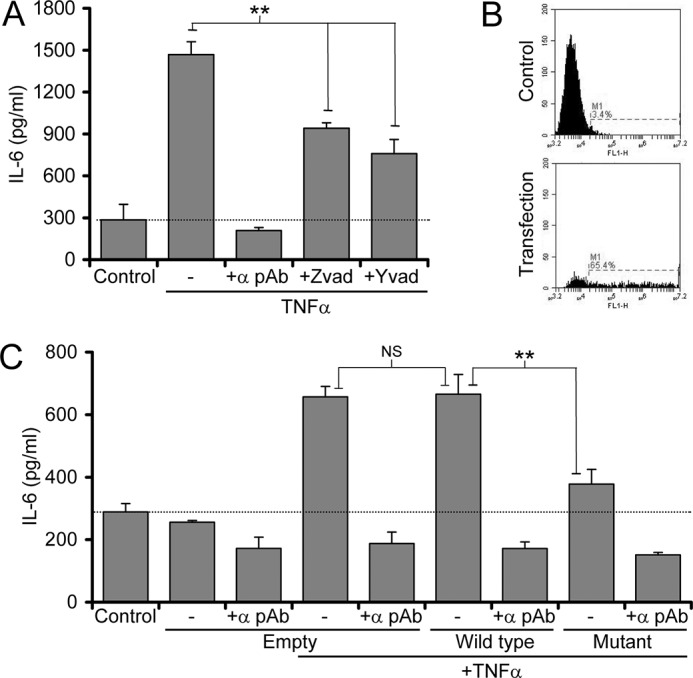
**Caspase-1 cleavage of IL-1R2 enables induction of IL-1α activity in stimulated ECs.** *A*, IL-1-dependent IL-6 production by HeLa cells incubated with and without IL-1α pAb with necrotic HUVEC lysates pretreated with TNFα with and without caspase inhibitors. *B*, flow cytometric analysis of HUVECs with and without transfection of a GFP expression plasmid. *C*, IL-1-dependent IL-6 production by HeLa cells incubated with and without IL-1α pAb with necrotic HUVEC lysates from cells transfected with WT or mutant IL-1R2 as indicated pretreated with and without TNFα. Data represent the mean ± S.E. of *n* = 3; **, *p* ≤ 0.01. *NS* = not significant.

IL-1R2 cleavage by caspase-1 is reported to cause its disassociation from pro-IL-1α, which subsequently enables calpain cleavage and activation (10). As no significant change in IL-1R2 protein level was found (Fig. 3, *D* and *E*), we postulated that caspase-1 cleavage of IL-1R2 could be responsible for the decreased association with IL-1α seen after EC stimulation. HUVECs were treated concurrently with TNFα and the pan-caspase inhibitor ZVAD-fluoromethyl ketone or the caspase-1-specific inhibitor YVAD-fluoromethyl ketone. Both inhibitors significantly reduced TNFα-induced IL-1α activity seen in necrotic lysates (Fig. 5*A*). Importantly, as all TNFα-induced IL-1 activity was IL-1α-specific, this excludes any effect of caspase inhibition on IL-1β activation. To confirm that caspase action increased IL-1α activity by promoting IL-1R2 cleavage, we expressed a form of IL-1R2 that cannot be cleaved by caspase-1 due to mutation of the key Asp residues (10). Using nucleofection, a HUVEC transfection efficiency of ∼62% was achieved (Fig. 5*B*). Transfection of wild-type IL-1R2 had no effect on TNFα-induced IL-1α activity (Fig. 5*C*). In contrast, expression of mutant IL-1R2 significantly reduced IL-1α activity (Fig. 5*C*). Importantly, transfection of an empty plasmid neither induced nor inhibited TNFα-induced IL-1α activity (Fig. 5*C*). Together, these experiments strongly suggest that cleavage of IL-1R2 by caspase-1 is an important step that enables the subsequent activation of IL-1α in necrotic EC lysates.

##### Necrotic EC Lysates Activate Adjacent Cells to a Proinflammatory State

**FIGURE 6.**
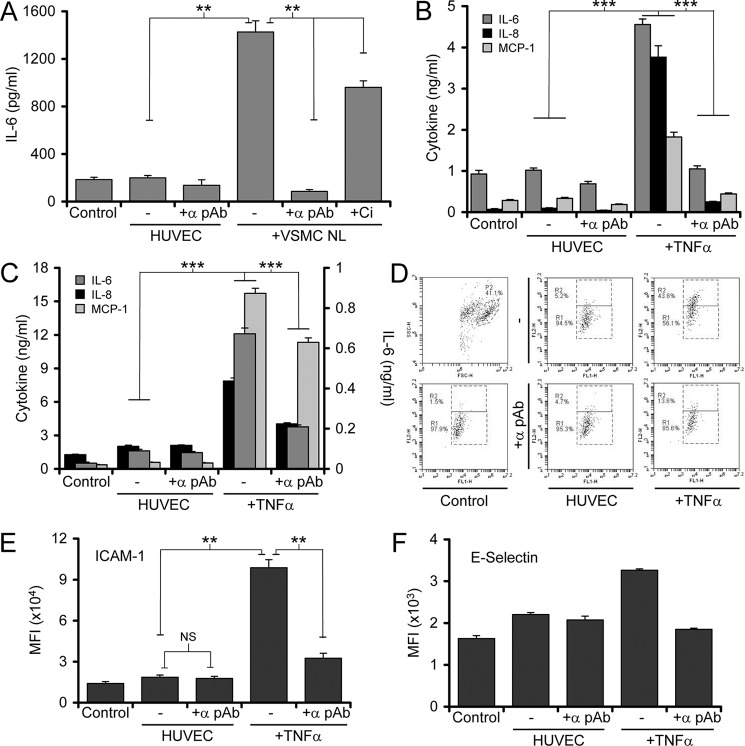
**Necrotic EC lysates activate adjacent cells to a proinflammatory state.** *A*, IL-1α activity within necrotic HUVEC lysates pretreated with and without calpeptin necrotic VSMC lysates, with and without IL-1α pAb during the priming step, or with and without calpeptin (Ci) during preparation of the necrotic HUVEC lysate. *B* and *C*, cytokine content of conditioned media from control VSMCs (*B*) or HUVECs (*C*) with and without calpeptin α pAb incubated with necrotic HUVEC lysates pretreated with and without calpeptin TNFα. Note the MCP-1 values in *C* plotted at 50%. *D*, FACs plots show that IL-1α-dependent increase in surface E-selectin expression after treatment with necrotic HUVEC lysates pretreated with and without TNFα. *E* and *F*, mean fluorescent intensities (*MFI*) of HUVEC populations stained for intercellular adhesion molecule 1 (*ICAM-1*; *E*) or E-selectin (*F*) analyzed by FACs after treatment as indicated with and without calpeptin α pAb. Data represent the mean ± S.D. of *n* = 3 (*A–C*) and 2 (*D–F*); **, *p* ≤ 0.01; ***, *p* ≤ 0.001. *NS* = not significant.

Given that necrotic EC-derived IL-1α drives graft rejection and that these data show that ECs need priming to activate IL-1α, we investigated the source of this initial stimulus. TNFα can be produced by ECs after IL-1, LPS, or TNFα treatment (11, 12). However, this requires time for transcription, translation, processing, and secretion. In contrast, as VSMCs can activate IL-1α upon necrosis without prior cytokine activation (Ref. 10 and Fig. 1*A*) whereas multiple other cell types can not (data not shown and Ref 10), we tested whether VSMC necrotic lysates could prime ECs. Treatment of HUVECs with necrotic VSMC lysates resulted in significant increases in active IL-1α in necrotic EC lysates (Fig. 6*A*), which was inhibited by neutralization of the VSMC-derived IL-1α during the priming step (Fig. 6*A*) or calpain inhibition during preparation of the HUVEC necrotic lysate (Fig. 6*A*). This suggests that VSMCs damaged during the perioperative period could provide active IL-1α that can prime ECs. Similarly, we tested whether necrotic EC lysates could activate adjacent ECs and VSMCs. Treatment of VSMCs (Fig. 6*B*) or HUVECs (Fig. 6*C*) with necrotic HUVEC lysates led to significant release of IL-6, IL-8, and MCP-1, which was dependent upon necrotic EC-derived IL-1α. In addition, HUVECs also showed IL-1α-dependent up-regulation of the cell surface adhesion molecules E-selectin and intercellular adhesion molecule 1 (*ICAM-1*; Fig. 6, *D–F*). Together these data indicate that interplay between damaged VSMCs and ECs could initiate activation of IL-1α in ECs, which in turn can activate adjacent vessel wall cells to effectively amplify inflammation.

## Discussion

Immune system activation protects organisms from pathogens able to challenge host fitness, usually by detecting pathogen-associated molecular patterns that are recognized as non-self by innate immune cells. However, immune activation also occurs without infection as a vital means of dealing with physical insults such as trauma, burns, tissue anoxia, or cytotoxic chemicals. This sterile inflammation is instigated by host-derived factors known as damage-associated molecular patterns that usually reside intracellularly but are released after cell damage. Conversely, immune activation by these endogenous factors also drives diseases such as arthritis, atherosclerosis, and graft rejection. Although IL-1α released from damaged ECs drives immune responses that cause arteriosclerosis and graft failure (6, 7), what controls IL-1α activity in ECs is unknown.

**FIGURE 7.**
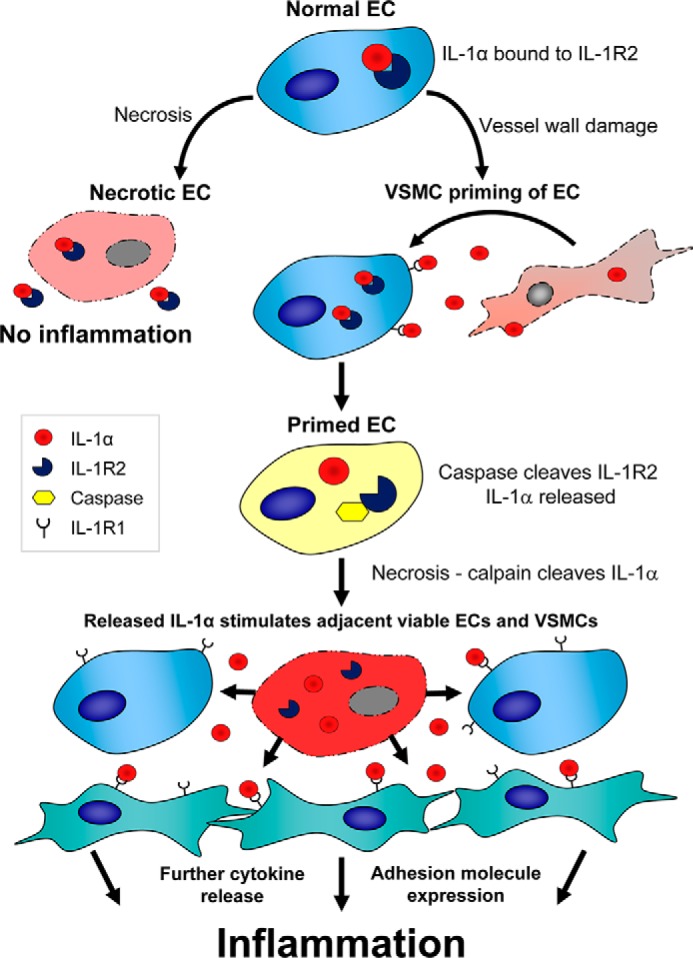
**Simplified diagram outlining the findings of the work.** We show that ECs that undergo necrosis without prior stimulation have no IL-1α activity. Priming with IL-1 or TNFα before necrosis induces caspase-1 cleavage of IL-1R2 and disassociation from IL-1α. ECs that subsequently undergo necrosis release calpain-cleaved fully active IL-1α that induces cytokine release and adhesion molecule expression in adjacent cells, thus amplifying inflammation.

We find that unstimulated ECs constitutively express small amounts of IL-1α protein but that it has no activity upon release from damaged cells. However, after prior activation with TNFα or IL-1, necrotic EC lysates show significantly increased IL-1α activity without any increase in IL-1α protein. Necrotic lysates made from unstimulated ECs contain only pro-IL-1α, whereas stimulated necrotic EC lysates contain calpain-cleaved mature IL-1α, which is much more active. Unstimulated ECs have cytosolic IL-1α that is co-localized and associated with IL-1R2, which protects it from calpain cleavage and activation. Stimulated ECs show reduced IL-1α/IL-1R2 interaction due to caspase cleavage of IL-1R2, which causes complex disassociation and enables calpain to cleave pro-IL-1α. Unstimulated ECs can be primed by damaged adjacent VSMCs that natively contain active IL-1α, whereas necrotic EC lysates from stimulated cells cause cytokine release and adhesion receptor up-regulation in adjacent viable VSMCs and ECs, thereby amplifying inflammation (outlined in Fig. 7). Thus, interplay between damaged ECs and other vessel wall cells leads to IL-1α activation that can drive subsequent Th17 responses and drive graft rejection.

IL-1 is a likely candidate to drive graft failure, as it can both induce inflammation and activate adaptive immunity. Indeed, inhibition of IL-1 signaling with IL-1RA or sIL-1R1 promotes survival in cardiac allografts (13), corneal grafts (14), and islet cell transplants (15) and also reduces graft *versus* host disease after bone marrow transplant (16). Furthermore, components of the inflammasomes upstream of IL-1 activation also show up-regulation during graft rejection (17). Although we currently do not understand how an intracellular form of IL-1R2 is expressed, it is present inside multiple cell types (10) including ECs. Using adenovirus-mediated transfer of sIL-1R2 to endothelial cells within cardiac allografts, Simeoni *et al.* (18) found decreased CD4+ cell infiltration and significantly increased graft survival, again supporting inhibition of IL-1 as a means of preventing graft rejection. IL-1 signaling typically results in production of IL-6, MCP-1, and IL-8. In particular, IL-1 and IL-6 strongly promote Th17 differentiation of T cells (19) and the subsequent IL-17 production that drives rejection (20). Furthermore, although MCP-1 is considered a classic chemokine for monocytes, it is also a powerful T cell chomoattractant (21). Indeed, Rao (7) reported significantly decreased T cell recruitment and IL-17 production *in vivo* after IL-1 blockade during vessel allograft rejection.

IL-1α is rarely found outside cells or in the circulation without cell death and as such has been described as an Alarmin, *i.e.* an endogenously derived DAMP. Although still contested (22), we and others find that IL-1α requires cleavage for full activity, with the proform showing low receptor affinity (9, 10). Although EC-derived IL-1α drives allograft rejection, our data show that IL-1α is uncleaved and inactive in unstimulated ECs due to its association with IL-1R2. ECs need an initial “insult” to generate active IL-1α such as pretreatment with TNFα or IL-1, which leads to the caspase-dependent release of IL-1R2 from pro-IL-1α. Caspase-1 is normally activated by the supramolecular inflammasomes that typically require an initial signal through Toll-like receptors (*e.g.* LPS) followed by a second stimulus (*e.g.* ATP, crystalline material) that leads to caspase-1 activation (23). However, some cell types only require the first toll-like receptor signal to activate caspase-1 (*e.g.* monocytes) (24). Therefore, as IL-1R1 and toll-like receptors share the same TIR domain and signaling pathway, IL-1 stimulation functionally equates to toll-like receptor ligation and thus could lead to caspase-1 activation in ECs. Two independent lines of evidence support the involvement of caspase-1 in this process; first, use of either a pan- or caspase-1-specific inhibitor reduces IL-1α activation after necrosis; second, expression of IL-1R2 with a mutation in the specific caspase-1 cleavage site acts as a dominant negative in ECs, resulting in less activation of IL-1α after necrosis. Interestingly, despite activation of caspase-1, we see no evidence of IL-1β activity in necrotic EC lysates, suggesting that ECs either do not express pro-IL-1β or that it is compartmentalized away from the caspase-1 activity.

These and previous data (6, 7) also suggest that the burden of injury hypothesis (3) and the immune modulation hypothesis (1) of graft rejection cannot be mutually exclusive. Necrotic ECs cannot activate IL-1α without initial damage to vessels that cause release of active IL-1α from VSMCs or other sources, However, once primed, ECs can subsequently activate IL-1α upon necrosis and in addition up-regulate adhesion molecules such as intercellular adhesion molecule 1 and E-selectin, leading to recruitment of leukocytes. Within this milieu of IL-1α- and IL-1-induced cytokines CD4 memory T cells will be pushed toward the Th17 lineage with production of IL-17 and instigate arteriosclerosis and graft rejection. This modulation of adaptive immunity can cause destruction of vascular wall cells and acute rejection. However, although effective immunosuppression prevents this acute response, chronic rejection still occurs, with progressive stenosis due to neointimal expansion causing tissue ischemia. Innumerous factors have been described to cause VSMC migration and proliferation in multiple models of neointima formation; however, what growth factors or cytokines cause this during graft arteriosclerosis is poorly understood. Interestingly, as the pathological changes remain confined to the donor tissue (literally stopping at the suture lines) and do not affect the host vasculature (25), this suggests that neointimal formation is dependent on cell-cell interactions rather than soluble mitogenic factors.

In conclusion, our data show that although EC-derived IL-1α is key in driving graft arteriosclerosis, it is inactive in unstimulated ECs due to binding to IL-1R2. ECs need priming via IL-1α derived from damaged VSMCs or TNFα to enable IL-1α activation upon necrosis, which leads to caspase-1-mediated cleavage of IL-1R2 and complex disassociation. In addition, necrotic EC-derived IL-1α causes inflammatory cytokine production and adhesion molecular up-regulation by adjacent ECs and VSMCs. These data help clarify the complex interplay between cell types in perioperatively damaged vessels that lead to IL-1-driven adaptive immunity and subsequent rejection. In addition, these data suggest that inhibition of inflammasomes, caspase-1, calpain, or IL-1α are potential therapeutic candidates to prevent arteriosclerosis and allograft rejection.

## Author Contributions

M. C. H. C. conceived and coordinated the study and wrote the paper. L. C. B. designed, performed, and analyzed the experiments. M. H. provided technical assistance and produced recombinant proteins. M. R. B. critically revised the manuscript for important intellectual content. All authors analyzed the results and approved the final version of the manuscript.

## Footnotes

2 The abbreviations used are:

VSMC: vascular smooth muscle cell
EC: endothelial cell
IL-1R2: interleukin-1 receptor-2
DAMP: damage-associated molecular pattern
Th17: T helper cell 17
HUVEC: human umbilical vein endothelial cell
pAb: polyclonal antibody.
